# Beyond the Concepts of Elder and Marginal in DCD Liver Transplantation: A Prospective Observational Matched-Cohort Study in the Italian Clinical Setting

**DOI:** 10.3389/ti.2023.11697

**Published:** 2023-09-07

**Authors:** Guido Fallani, Alberto Stocco, Antonio Siniscalchi, Marta Velia Antonini, Adriano Pasquale Stella, Alessio Amato, Enrico Prosperi, Laura Turco, Maria Cristina Morelli, Matteo Cescon, Matteo Ravaioli

**Affiliations:** ^1^ Department of Hepatobiliary Surgery and Transplantation, Policlinico di Sant’Orsola, Istituto di Ricovero e Cura a Carattere Scientifico, Azienda Ospedaliero-Universitaria di Bologna, Bologna, Italy; ^2^ Department of Transplant Intensive Care Unit, Policlinico di Sant’Orsola, Istituto di Ricovero e Cura a Carattere Scientifico, Azienda Ospedaliero-Universitaria di Bologna, Bologna, Italy; ^3^ Ospedale “Maurizio Bufalini”—Azienda Unità Sanitaria Locale Romagna, Cesena, Italy; ^4^ Department of Biomedical, Metabolic and Neural Sciences, University of Modena and Reggio Emilia, Modena, Italy; ^5^ Department of Internal Medicine for the Treatment of Severe Organ Failure, Policlinico di Sant’Orsola, Istituto di Ricovero e Cura a Carattere Scientifico, Azienda Ospedaliero-Universitaria di Bologna, Bologna, Italy; ^6^ Department of Medical and Surgical Sciences (DIMEC), University of Bologna, Bologna, Italy

**Keywords:** liver transplantation, elderly donors, donation after circulatory determination of death, donation after brainstem death, liver transplantation outcomes

## Abstract

Donation after circulatory determination of death (DCD) is a valuable strategy to increase the availability of grafts for liver transplantation (LT). As the average age of populations rises, the donor pool is likely to be affected by a potential increase in DCD donor age in the near future. We conducted a prospective cohort study to evaluate post-transplantation outcomes in recipients of grafts from elderly DCD donors compared with younger DCD donors, and elderly donors after brainstem determination of death (DBD). From August 2020 to May 2022, consecutive recipients of deceased donor liver-only transplants were enrolled in the study. DCD recipients were propensity score matched 1:3 to DBD recipients. One-hundred fifty-seven patients were included, 26 of whom (16.6%) were transplanted with a DCD liver graft. After propensity score matching and stratification, three groups were obtained: 15 recipients of DCD donors ≥75 years, 11 recipients of DCD donors <75 years, and 28 recipients of DBD donors ≥75 years. Short-term outcomes, as well as 12 months graft survival rates (93.3%, 100%, and 89.3% respectively), were comparable among the groups. LT involving grafts retrieved from very elderly DCD donors was feasible and safe in an experienced high-volume center, with outcomes comparable to LTs from younger DCD donors and age-matched DBD donors.

## Graphical Abstract

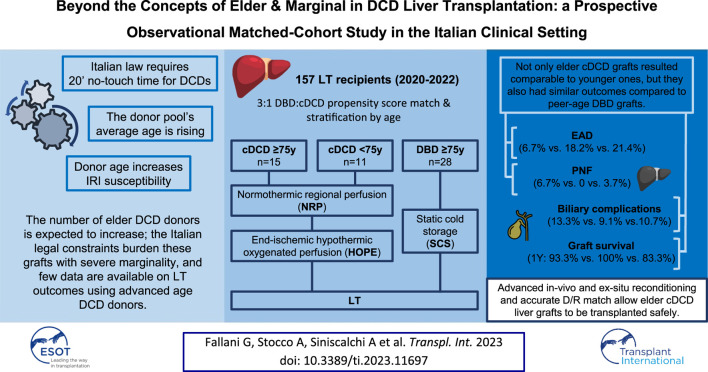

## Introduction

Liver transplantation (LT) is considered the treatment of choice for patients with end-stage liver disease. The inclusion of extended criteria donors (ECDs) and donors after circulatory determination of death (DCD) is growing in the attempt to address the critical gap between donors and recipients. DCD donors are a valuable source of grafts, even if concerns have been raised about potentially impaired outcomes related to prolonged warm ischemia time (WIT). Nevertheless, several recent studies have described acceptable results after transplantation involving those donors [1, 2].

According to Italian law [3, 4], death can only be declared after 20 min of lack of any cardiac electrical activity. A strategy of *in-situ* normothermic regional perfusion (NRP) [5]—aimed to interrupt the prolonged ischemia and to maintain a near-physiologic environment during retrieval [6]—is considered mandatory in the Italian scenario. Moreover, to further mitigate ischemia-reperfusion injury (IRI), most Italian transplant centers also implement end-ischemic hypothermic oxygenated perfusion (HOPE) on DCD liver grafts [7].

The constant increase in the average age of the general population also implies a subsequent change in the demography of the organ donor pool. This phenomenon is most likely to affect the pool of DCD donors too, and data on LT from advanced-age DCD donors are emerging in the literature [8–10].

Even if donor age impacts the outcomes of LT [11–13], the acceptance of older DBD donor grafts could be effective in selected recipients [14]. Thus, this study aims to compare the outcomes of LTs involving elder DCD donors with both younger DCD donors and elder DBD donors in the specific context of an experienced Italian transplant center.

## Methods

We conducted a single center prospective observational cohort study including consecutive recipients of liver-only transplantation of controlled DCD (cDCD) donors [15, 16] from August 2020 to May 2022. Based on pre-transplant donor and recipient characteristics, cDCD liver recipients underwent propensity score matching (PSM) with recipients of liver-only transplantation from DBD donors in a 1:3 DCD:DBD ratio. The study population was stratified by age to compare outcomes of grafts from younger cDCD donors (<75 years-old) and elder cDCD donors (≥75 years-old), with elder DBD donors (≥75 years-old).

Informed consent was obtained from all the recipients. The study was approved by the Institutional Review Board (Comitato Etico—Area Vasta Emilia Centro, CE-AVEC, protocol no. 895/2021/Oss/AOUBo).

### Donor Management and Procurement

The technique of abdominal organ procurement in cDCD donors in Italy has been previously described in detail [17], and the timeline of events is reported in Supplementary Figure S1.

After circulatory arrest and no-touch time, NRP is initiated, targeting lactate clearance, pH normalization, normocapnia, and avoidance of hyperoxemia; hemoglobin concentration is maintained above 8 g/dL and hyperglicemia is corrected to facilitate organ resuscitation. Since NRP starts every 30′, blood gas analysis and liver enzyme measurement are performed throughout extracorporeal perfusion. NRP is maintained for at least 60–90 min to assess the organ functional recovery; the criteria of viability include fWIT<60 min, pH normalization and stability, progressive lactate decrease, and SGOT/SGPT decrease (usually evident after 30 min of NRP). When the metabolic and perfusion profiles of the donors are considered optimal, the surgical procedure is initiated: first, liver (and kidney) biopsies are obtained, then the warm dissection phase of the procurement is performed. In our practice, liver biopsy is a cornerstone to assess organ viability, as the presence of extensive lobular necrosis represents a contraindication to further proceed with retrieval.

NRP ends with *in-situ* cold preservation (ISP): once the organs are retrieved, they are put in static cold storage (SCS) to be transported to the transplant center where bench surgery is performed, and HOPE is implemented until implantation.

Conversely, liver grafts from DBD donors were retrieved with standard technique and preserved with SCS until implantation.

### Outcome Definitions and Measures

**FIGURE 1:**
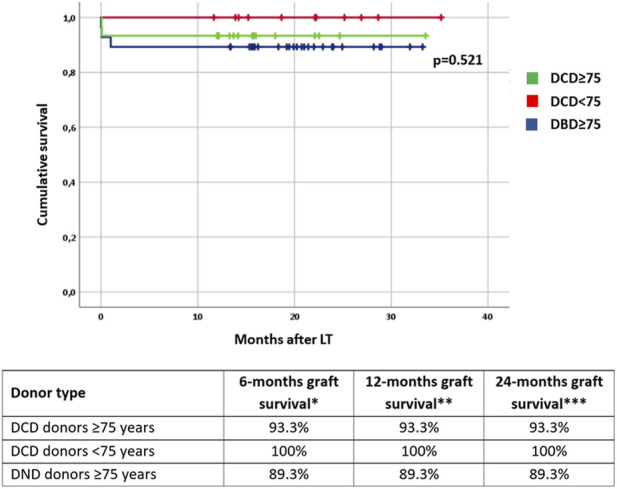
Graft survival rates stratified by donor type and age. DCD: donation after circulatory determination of death; DBD, donation after brainstem death. * number of patients at risk: 54; ** number of patients at risk: 54; *** number of patients at risk: 14.

For DCD donors, total WIT (tWIT) was defined as the timeframe occurring between WLST and NRP initiation, while functional WIT (fWIT) was defined as the timeframe between hypotension (systolic arterial pressure below 50 mmHg) or desaturation (peripheral oxygen saturation below 70%)—whichever occurring first—and NRP initiation. Cold preservation time (CPT) was defined as the interval from aortic cross-clamping/ISP and portal reperfusion upon LT, thus including both SCS and HOPE duration (Figure 1).

Complications were defined as any event deviating from the expected postoperative course that does not imply failure to cure [18]. For each patient, the postoperative complications were graded according to the Clavien-Dindo classification [18] and summarized through the Comprehensive Complication Index (CCI®) [19]; major complications were defined as Clavien-Dindo grade ≥3A.

Post-reperfusion syndrome (PRS) was defined according to Aggarwal et al. [20] Primary non-function (PNF) of the graft was defined according to the Organ Procurement and Transplantation Network (OPTN) criteria [21], while early allograft dysfunction (EAD) was defined according to the criteria proposed by Olthoff et al. [22] Severe acute kidney injury was defined according to the Kidney Disease: Improving Global Outcomes (KDIGO) clinical practice guideline [23].

### Statistical Analysis

Qualitative variables were reported as absolute values and percentages, whereas quantitative variables were reported as median values and IQR or mean ± SD as appropriate. Univariate analysis was performed using Pearson’s chi-squared or Fisher’s exact test for categorical variables, depending on the sample size, and with Student’s t-test or Mann-Whitney U test for continuous variables, depending on their distribution.

PSM was performed through logistic regression analysis to adjust for clinically confounding factors between groups, including donor age, recipient age at the time of transplantation, MELD score, and hepatocellular carcinoma (HCC) as an indication for LT. DCD and DBD recipients were matched in a 1:3 ratio with the closest estimated propensity. Survival curves were plotted with the Kaplan-Meier estimators and compared through the log-rank test.

Differences of *p* < 0.05 were considered significant.

All the statistical analyses were performed using IBM SPSS, version 26.0 (IBM Corporation—Armonk, NY).

## Results

From August 2020 to May 2022, 183 liver grafts were offered for transplantation to the Department of Hepatobiliary Surgery and Transplantation of Policlinico Sant’Orsola in Bologna. Of these, 30 (16.4%) came from DCD donors: four were declined, with an acceptance rate of 86.7%. Two livers were discarded due to a combination of prolonged fWIT, and poor liver and metabolic parameters during NRP (as per our aforementioned criteria). The remaining two livers were refused as malignancy was detected during the retrieval procedure. The remaining 153 liver grafts offered for transplantation (83.6%) came from DBD donors. Of these, 12 were discarded for marginality upon senior transplant surgeon judgment, with an acceptance rate of 92.2%. Other 10 liver grafts were allocated for multiorgan transplantation or re-transplantation and thus excluded from the study.

The final population included in the study consisted of 157 recipients, 131 of which (83.4%) were transplanted with grafts from DBD donors, and 26 (16.6%) with grafts from cDCD donors.

### Donor and Recipient Pre- and Post-operative Characteristics

Compared with DBD donors, DCDs had higher median age (75 years vs. 63 years, *p* = 0.018); notably, most DCD donors were 75 years or older. Recipients of DCD liver grafts had lower MELD scores (10 vs. 15, *p* < 0.001), and were more often transplanted due to HCC (69.2% vs. 41.2%, *p* = 0.009). Full demographic and clinical characteristics of recipients and donors are summarized in Table 1.

**TABLE 1:** Baseline preoperative recipients’ characteristics and donor characteristics before and after propensity score matching.

Variables	Before PSM			After PSM		
	DCD-LT (*n* = 26)	DBD-LT (*n* = 131)	*p*	DCD-LT (*n* = 26)	DBD-LT (*n* = 78)	*p*
Recipient age in years, median [IQR]	61 [56–64]	58 [53–64]	0.214	61 [56–64]	59 [54–65]	0.435
Recipient BMI in kg/m^2^, median [IQR]	23.8 [22.3–29.2]	26 [23.1–28.1]	0.340	23.8 [22.3–29.2]	25.5 [22.8–28.1]	0.538
Indication for LT						
Hepatocellular carcinoma, n (%)	18 (69.2)	54 (41.2)	**0.009**	18 (69.2)	41 (52.6)	0.137
Virus-related cirrhosis, n (%)	10 (38.5)	52 (39.7)	0.906	10 (38.5)	31 (39.7)	0.908
Alcohol-relates cirrhosis, n (%)	8 (30.8)	52 (39.7)	0.392	8 (30.8)	31 (39.7)	0.413
NAFLD, n (%)	3 (11.5)	24 (18.3)	0.572	3 (11.5)	11 (14.1)	1
Cholestatic liver disease, n (%)	3 (11.5)	17 (13)	1	3 (11.5)	12 (15.4)	0.756
Acute liver failure, n (%)	0	6 (4.6)	0.590	0	2 (2.6)	1
Other, n (%)	4 (15.4)	27 (20.6)	0.541	4 (15.4)	15 (19.2)	0.766
Previous abdominal surgery, n (%)	17 (65.4)	76 (58.5)	0.511	17 (65.4)	51 (66.2)	0.937
Previous liver resection, n (%)	5 (19.2)	10 (7.6)	0.077	5 (19.2)	8 (10.3)	0.303
TIPSS, n (%)	3 (11.5)	12 (7.9)	0.463	3 (11.5)	3 (3.8)	0.163
Portal thrombosis, n (%)	4 (15.4)	24 (18.3)	1	4 (15.4)	12 (15.4)	1
Platelet count *10^3^/µL, median [IQR]	103 [63–161]	72 [46–133]	0.140	103 [63–161]	96 [61–154]	1
MELD at transplant, median [IQR]	10 [8–14]	15 [10–24]	**<0.001**	10 [8–14]	12 [9–15]	0.297
Recipient comorbidities						
Diabetes mellitus, n (%)	10 (40)	39 (29.8)	0.321	10 (40)	22 (28.2)	0.267
Cardiovascular disease, n (%)	3 (11.5)	28 (21.4)	0.250	3 (11.5)	16 (20.5)	0.390
Respiratory disease, n (%)	6 (23.1)	26 (19.8)	0.709	6 (23.1)	15 (19.2)	0.672
Renal disease, n (%)	3 (11.5)	18 (13.7)	1	3 (11.5)	7 (9)	0.708
Donor age in years, median [IQR]	75 [64–78]	63 [50–75]	**0.018**	75 [64–78]	69 [56–79]	0.367
Donor BMI in kg/m^2^, median [IQR]	26.2 [23.1–29.8]	25.7[23.8–27.8]	0.797	26.2 [23.1–29.8]	25.9 [24.2–29.1]	0.901

DCD-LT, donation after circulatory determination of death liver transplantation; DBD-LT, donation after brainstem death liver transplantation; IQR, interquartile range; BMI, body mass index; NAFLD, nonalcoholic fatty liver disease; TIPSS, transjugular intrahepatic portosystemic shunt; MELD, model for end-stage liver disease.

Bold values highlight statistical significance.

After PSM, the population resulted in 26 DCD donors and 78 DBD donors; the subsequent analysis demonstrated the comparability of baseline characteristics (Table 1).

DCD grafts had a median tWIT of 45 min and a median fWIT of 40 min, with a median timeframe of 6 min from death declaration to NRP initiation. NRP had a median duration of 209 min and end-ischemic HOPE had a median duration of 105 min. No significant differences have been observed between elder and younger DCD donors in terms of tWIT, fWIT, NRP duration, metabolic and functional parameters during NRP (pH, lactates, SGOT, SGPT), bioptic findings, or HOPE duration. These results are summarized in Supplementary Table S1. Grafts from DCD donors underwent shorter CPT (345 vs. 388 min, *p* = 0.010) and shorter duration of transplant (430 vs. 470 min, *p* = 0.040).

The analysis of postoperative data showed that recipients of livers from DCD and DBD donors had comparable results in terms of surgical complications, length of hospital stay, and graft function (Table 2).

**TABLE 2:** Procedural and outcome data after propensity score matching.

Variables	DCD-LT (n = 26)	DBD-LT (*n* = 78)	*p*
Cold preservation time in minutes, median [IQR]	345 [314–393]	388 [344–473]	**0.010**
Reperfusion syndrome, n (%)	2 (7.7)	2 (2.7)	0.260
Transplant duration in minutes, median [IQR]	430 [381–493]	470 [429–523]	**0.040**
ICU stay in days, median [IQR]	4 [3–5]	3 [2–5]	0.116
Peak SGOT (POD 1–7) in U/L, median [IQR]	347 [259–1,026]	566 [289–1,361]	0.363
Peak SGPT (POD 1–7) in U/L, median [IQR]	495 [168–854]	519 [272–1,057]	0.416
Post-operative infectious complications, n (%)	7 (26.9)	20 (25.6)	0.896
Severe acute kidney injury, n (%)	1 (4)	4 (5.1)	1
Respiratory failure, n (%)	0	2 (2.6)	1
Post-operative haemorrhage, n (%)	0	1 (1.3)	1
Reintervention, n (%)	1 (4)	8 (10.3)	0.546
Hepatic artery thrombosis, n (%)	0	1 (1.3)	1
12 months biliary complications, n (%)	3 (11.5)	7 (9.1)	0.710
30 days acute cellular rejection, n (%)	0	3 (3.9)	0.570
90 days mortality, n (%)	0	2 (2.6)	1
Early allograft dysfunction, n (%)	3 (11.5)	17 (21.8)	0.250
Primary graft non function, n (%)	1 (4)	3 (3.9)	1
Re-transplantation, n (%)	1 (4)	5 (5.3)	0.678
Major complications, n (%)	4 (15.4)	16 (20.5)	0.566
Comprehensive Complication Index^®^, 75th percentile	29.6	30.8	0.487
Hospital stay in days, median [IQR]	15 [13–23]	15 [11–23]	0.919

DCD-LT, donation after circulatory determination of death liver transplantation; DBD-LT, donation after neurological determination of death liver transplantation; IQR, interquartile range; NRP, normothermic regional perfusion; HOPE, hypothermic oxygenated perfusion; ICU, intensive care unit; SGOT, serum glutamic oxaloacetic transaminase; SGPT, serum glutamic pyruvic transaminase; POD, postoperative day.

Bold values highlight statistical significance.

### Post-Transplant Outcomes According to Donor Type and Donor Age

After stratification by age, three subgroups were identified: 15 recipients of DCD donors ≥75 years, 11 recipients of DCD donors <75 years, and 28 recipients of DBD donors ≥75 years. No significant differences were evident in terms of CPT, surgical complications, length of hospital stay, and graft function. One recipient of an elder DCD donor experienced PNF and was successfully retransplanted. Amongst the elder DBD recipients two have been retransplanted due to PNF in one case and hepatic artery thrombosis in the other; another recipient of an elder DBD donor died a few hours after LT due to massive myocardial infarction. Altogether, six patients developed biliary complications during the follow-up period (with comparable rates between groups), all consisting of anastomotic strictures with successful endoscopic management. The results are summarized in Table 3. Patients were followed up for a median of 19 months [IQR: 14–24 months] without any significant difference in terms of graft survival for the three subgroups (Figure 1).

**TABLE 3:** Procedural and outcome data after propensity score matching and stratification by donor type and age.

Variables	DCD≥75 [group 1] (*n* = 15)	DCD<75 [group 2] (*n* = 11)	DBD≥75 [group 3] (*n* = 28)	*p* (1 vs. 2)	*p* (1 vs. 3)
Cold preservation time in minutes, median [IQR]	335 [300–390]	350 [320–400]	360 [340–405]	0.336	0.097
Reperfusion syndrome, n (%)	2 (13.3)	0	1 (3.6)	0.492	0.275
Transplant duration in minutes, median [IQR]	425 [383–486]	480 [360–505]	469 [419–503]	0.568	0.221
ICU stay in days, median [IQR]	3 [3–5]	4 [3–5]	3 [2–5]	0.576	0.467
Peak SGOT (POD 1–7) in U/L, median [IQR]	429 [298–1,010]	305 [253–1,399]	478 [248–1,218]	0.494	0.904
Peak SGPT (POD 1–7) in U/L, median [IQR]	501 [159–825]	292 [176–1,572]	555 [210–1,007]	0.913	0.775
Post-operative infectious complications, n (%)	5 (33.3)	2 (18.2)	9 (32.1)	0.679	0.938
Severe acute kidney injury, n (%)	0	1 (9.1)	0	0.440	—
Respiratory failure, n (%)	0	0	0	—	—
Post-operative haemorrhage, n (%)	0	0	0	—	—
Reintervention, n (%)	1 (6.7)	0	3 (10.7)	0.874	0.909
Hepatic artery thrombosis, n (%)	0	0	1 (3.7)	—	1
12 months biliary complications, n (%)	2 (13.3)	1 (9.1)	3 (10.7)	0.774	0.807
30 days acute cellular rejection, n (%)	0	0	2 (7.4)	—	0.530
90 days mortality, n (%)	0	0	1 (3.7)	—	1
Early allograft dysfunction, n (%)	1 (6.7)	2 (18.2)	6 (21.4)	0.556	0.391
Primary graft non function, n (%)	1 (6.7)	0	1 (3.7)	0.874	1
Re-transplantation, n (%)	1 (6.7)	0	2 (7.4)	0.874	0.956
Major complications, n (%)	3 (20)	1 (9.1)	6 (21.4)	0.614	1
Comprehensive Complication Index^®^, 75th percentile	29.6	29.6	30.5	0.979	0.612
Hospital stay in days, median [IQR]	15 [13–23]	13 [12–23]	17 [13–24]	0.465	0.798

DCD-LT, donation after circulatory determination of death liver transplantation; DBD-LT, donation after neurological determination of death liver transplantation; IQR, interquartile range; NRP, normothermic regional perfusion; HOPE, hypothermic oxygenated perfusion; ICU, intensive care unit; SGOT, serum glutamic oxaloacetic transaminase; SGPT, serum glutamic pyruvic transaminase; POD, postoperative day.

## Discussion

The average age of donors in Italy is continuously increasing, with the median age rising from 57.7 in 2012 to 60.9 years in 2021. The number of donors over the age of 80 is also increasing, representing—to date—a consistent portion of the donor pool (13.5% in 2021). In parallel, the mean age of cDCD donors increased to 67 years, with 6% of donations coming from octogenarians. In the hypothesis of a steady trend, a progressive increase in elderly cDCD donors is expected, possibly representing an additional opportunity for transplant candidates, especially for those at greater risk of drop-out or death while on the waiting list.

We reported the first Italian data involving a relatively large number of elder DCD donors; in particular, as far as we know, the 75 years age cut-off that we considered is higher than any other previously published and outreaches the eldest age reported in many reports on elderly DCD donors [8–10].

This study aimed to investigate whether utilizing very elderly DCD donors allows them to achieve comparable outcomes to younger DCD donors and peer-age DBD donors.

Since DCD in the Italian scenario entails severe ischemic burden on liver grafts, these donors have long been considered “marginal”. Nevertheless, recent literature demonstrated a progressive alignment of post-transplant outcomes to those of DBD donors in terms of graft function and graft and recipient survival [24–26]. These findings may suggest that an incremental volume and expertise of the transplant team play a significant role in contributing to the success of DCD liver transplantation. Furthermore, some reports showed that accepting DCD donors or elderly donors, despite their perceived marginality, can significantly improve the survival of selected recipients [27–29].

Our results appear to be consistent with this recent evidence. Although recipients of DCD liver grafts are characterized by a lower MELD score (10 vs. 15, *p* < 0.001), and a higher prevalence of HCC as a primary indication to transplant (69.2% vs. 41.2%, *p* = 0.009), post-operative outcome results were comparable after adequate minimization of existing biases.

Despite the requirement for PSM, it is important to mention that the different utilization of DCD and DBD liver grafts in our case series reflects the contemporary trends from medical literature and the higher individual transplant benefit for patients at high risk of drop-out from the waiting list [27–29]. Consistently with this evidence, we usually match DCD donors with recipients who, despite stable liver function, have a high-risk of drop-out from the waiting list due to either oncological risk (e.g., recurrent or down-staged HCC) or infectious risk (e.g., recurrent cholangitis); donor age has little influence in our allocation algorithm, given that DCD donors are preferably accepted for patients with stable liver function, which can more easily tolerate the increased ischemic burden. As a result, outcomes of LT for both DCD and DBD donors not only appeared comparable after PSM, but they also showed similar results after stratification by age and type of donation. Specifically, recipients of very elderly DCD donors had homogenous results in terms of CPT, surgical complications, hospitalization, and graft function, compared to recipients of same-age DBD and younger DCD donors. Moreover, the rate of biliary complications was acceptable in all subgroups despite donors being at high risk, conversely to the previously reported data [30–32]. In our opinion, this resulted from the extensive use of HOPE for DCD grafts pre-conditioning, and from shorter CPT (345 min vs. 388 min, *p* = 0.010).

Considering the strict Italian legislation, the tWIT liver grafts are exposed to are strongly conditioned by the requirement of a 20-minute-long standoff period before NRP initiation. The outcomes of DCD liver transplantation observed in our cohort, besides being consistent with DBD transplantation, are also the result of meticulous donor management, coupling advanced strategies of *in-situ* and *ex-situ* perfusion strategies, and accurate, tailored donor-recipient matching. The improvement in donor and recipient management to minimize tWIT and CPT, starting with routine use of end-ischemic HOPE for DCD liver grafts, showed promising results in preventing and mitigating ischemic insults, and related post-transplant ischemia-reperfusion injury, ultimately leading to satisfactory results [33–36].

We can also assume that the strict Italian legislation [3, 4] aimed to overguarantee the respect of the so-called “dead-donor-rule”—played a major role in forcing transplant teams to pursue accurate management of both DCD donors and grafts in order to overcome the imposed procedural limitations.

### Limitations

This study has some limitations. First, the need for case-matching, as well as the limited number of patients in the derived subgroups, affected our ability to draw definite conclusions. Nevertheless, the relatively limited size of the study group reflects a hopefully initial experience with very elderly DCD donors. The short duration of the study implied a limited follow-up for the patients with a late enrolment, although a minimum twelve-month follow-up was provided for all the included recipients. Finally, in this study, we compared grafts from elderly cDCD donors—exposed to extensive reconditioning through NRP and end-ischemic HOPE—with ECD liver grafts (at least according to donor age) from DBD donors, which have been implanted without advanced perfusion strategies. This approach might be considered a bias, as DBD grafts can benefit from HOPE too [35, 36], but it reflects the state of the art in DCD liver transplantation in Italy. In fact, under Italian legal and procedural circumstances, this approach enabled the safe transplant of organs potentially carrying a relevant ischemic burden. Moreover, its extensive use also achieved comparable outcomes between cDCD grafts and ECD grafts, with the latter still being transplanted without HOPE in the majority of transplant centers worldwide.

### Conclusion

According to the preliminary results of our single center experience, the inclusion of very elderly cDCD donors in liver transplantation programs might provide acceptable outcomes, comparable to those achieved with younger cDCD donors, and with same-age DBD donors. With donor management and graft allocation and reconditioning becoming more and more accurate as further experience and evidence accumulate, the ability of clinicians to achieve optimal utilization and to improve transplantation outcomes for DCD grafts will be enhanced, overcoming existing concerns about these donors’ perceived marginality.

## Data Availability

The data that support the findings of this study are available upon reasonable request from the corresponding author.

## Supplementary Material

Supplementary Figure S1 Timeline of events preceding and following declaration of death with cardiocirculatory criteria Maastricht category 3 controlled donors. Agonal phase and fWIT start with hypotension (mean arterial pressure dropping below 50 mmHg) or desaturation (peripheral oxygen saturation dropping below 50%), whichever occurring first; tWIT and fWIT refer to the potential donor. NRP interrupts ischemic time. CIT refers to the graft; the time the graft is treated with hope is not to be considered functionally ischemic, despite the lack of hematic perfusion, as the liver is oxygenated. Times not to scale. WLST, withdrawal of life sustaining treatments; tWIT, total warm ischemia time; fWIT, functional warm ischemia time (hypoxemic and/or hypotensive); NRP, normothermic regional perfusion; ISP, in situ-preservation; SCS, static cold storage; CPT, cold preservation time; HOPE, hypothermic oxygenated perfusion; LT, liver transplantation.

## Abbreviations

aWIT, asystolic Warm Ischemia Time; CCI®, Comprehensive Complication Index; CPT, Cold Preservation Time; DBD, Donors after Brainstem Death; DCD, Donation after Circulatory determination of Death; EAD, Early Allograft Dysfunction; fWIT, functional Warm Ischemia Time; HCC, HepatoCellular Carcinoma; HOPE, Hypothermic Oxygenated PErfusion; IQR, Inter-Quartile Range; IRI, Ischaemia-Reperfusion Injury; ISP, In-Situ Perfusion; LT, Liver Transplant; MAP, Mean Arterial Pressure; MELD, Model for End Stage Liver Disease; NRP, Normothermic Regional Perfusion; OPTN, Organ Procurement and Transplantation Network; PNF, Primary Non Function; PRS, Post Reperfusion Syndrome; PSM, Propensity Score Match; SCS, Static Cold Storage; tWIT, total Warm Ischemia Time; WIT, Warm Ischemia Time.
