# Supplementary material for: Beyond the Concepts of Elder and Marginal in DCD Liver Transplantation: A Prospective Observational Matched-Cohort Study in the Italian Clinical Setting
Source: Transpl Int. 2023 Sep 7;36:11697. doi: 10.3389/ti.2023.11697 (PMC10511003; doi:10.3389/ti.2023.11697)
Supplement: Supplementary file 1 [file Table1.docx]

| **Variables** | **DCD-LT**  **(n=26)** | **DCD≥75**  **(n=15)** | **DCD<75**  **(n=11)** | **p**  **(≥75 vs. <75)** |
| --- | --- | --- | --- | --- |
| tWIT in minutes, median [IQR] | 45 [40-52] | 48 [40-53] | 43 [37-51] | 0.311 |
| fWIT in minutes, median [IQR] | 40 [38-48] | 40 [38-51] | 40 [35-46] | 0.566 |
| Time from death declaration to NRP start in minutes, median [IQR] | 6 [4-8] | 5 [4-7] | 6 [4-12] | 0.356 |
| NRP duration in minutes, median [IQR] | 209 [171-236] | 204 [174-236] | 218 [163-244] | 0.421 |
| HOPE duration in minutes, median [IQR] | 105 [63-130] | 120 [85-153] | 70 [60-113] | 0.054 |
| pH t_0_, median [IQR] | 6.99  [6.94-7.13] | 6.99  [6.94-7.14] | 7.03  [6.8-7.12] | 0.814 |
| Lactic acid t_0_ in mEq/l, median [IQR] | 10.9 [9.2-12.2] | 10.7 [8.8-11.9] | 11.2 [9.9-12.5] | 0.481 |
| SGOT t_0_ in UI/l, median [IQR] | 56 [36-139] | 79 [48-239] | 45 [31-60] | 0.194 |
| SGPT t_0_ in UI/l, median [IQR] | 52 [24-135] | 53 [28-141] | 37 [21-73] | 0.276 |
| pH t_1_, median [IQR] | 7.36  [7.29-7.39] | 7.37  [7.31-7.41] | 7.30  [7.19-7.44] | 0.226 |
| Lactic acid t_1_ in mEq/l, median [IQR] | 7.6 [6.4-10.4] | 7.1 [6.1-10.3] | 8.3 [6.7-10.6] | 0.678 |
| SGOT t_1_ in UI/l, median [IQR] | 63 [44-435] | 83 [54-485] | 47 [28-130] | 0.078 |
| SGPT t_1_ in UI/l, median [IQR] | 66 [30-162] | 34 [143-384] | 52 [28-73] | 0.146 |
| pH t_2_, median [IQR] | 7.39  [7.30-7.43] | 7.35  [7.30-7.44] | 7.40  [7.29-7.43] | 0.906 |
| Lactic acid t_2_ in mEq/l, median [IQR] | 6.1 [5-10] | 6.5 [5-10] | 6.1 [4.7-10.4] | 0.533 |
| SGOT t_2_ in UI/l, median [IQR] | 68 [47-335] | 209 [51-740] | 58 [38-213] | 0.187 |
| SGPT t_2_ in UI/l, median [IQR] | 74 [30-243] | 115 [29-378] | 63 [30-134] | 0.424 |
| Bioptic findings |  |  |  |  |
| Microsteatosis in %, median [IQR] | 1 [0-5] | 0 [0-5] | 5 [0-5] | 0.164 |
| Macrosteatosis in %, median [IQR] | 1 [0-5] | 0 [0-5] | 3 [0-5] | 0.559 |
| Lobular necrosis in %, median [IQR] | 0 [0-1] | 0 [0-1] | 0 [0-2] | 0.567 |
| Portal fibrosis – Ishak score, n (%) | 0 – 1 (3.8)  1 – 9 (34.6)  2 – 14 (53.8)  3 – 2 (7.7) | 0 – 0  1 – 6 (40)  2 – 8 (53.3)  3 – 1 (6.7) | 0 – 1 (9.1)  1 – 3 (27.3)  2 – 6 (54.5)  3 – 1 (9.1) | 0.635 |

**Supplementary Table 1.** Metabolic, functional, and histological parameters during in-situ and ex-situ perfusion of DCD donors. **t_0_** = NRP start, **t_1_** = 30’ after NRP start, **t_2_** = 60’ after NRP start. IQR: interquartile range; tWIT: total warm ischemia time; fWIT: functional warm ischemia time; NRP: normothermic regional perfusion; HOPE: hypothermic oxygenated perfusion; SGOT: serum glutamic oxaloacetic transaminase; SGPT: serum glutamic pyruvic transaminase.
