# Supplementary figures and images for: Beyond the Concepts of Elder and Marginal in DCD Liver Transplantation: A Prospective Observational Matched-Cohort Study in the Italian Clinical Setting
Source: Transpl Int. 2023 Sep 7;36:11697. doi: 10.3389/ti.2023.11697 (PMC10511003; doi:10.3389/ti.2023.11697)

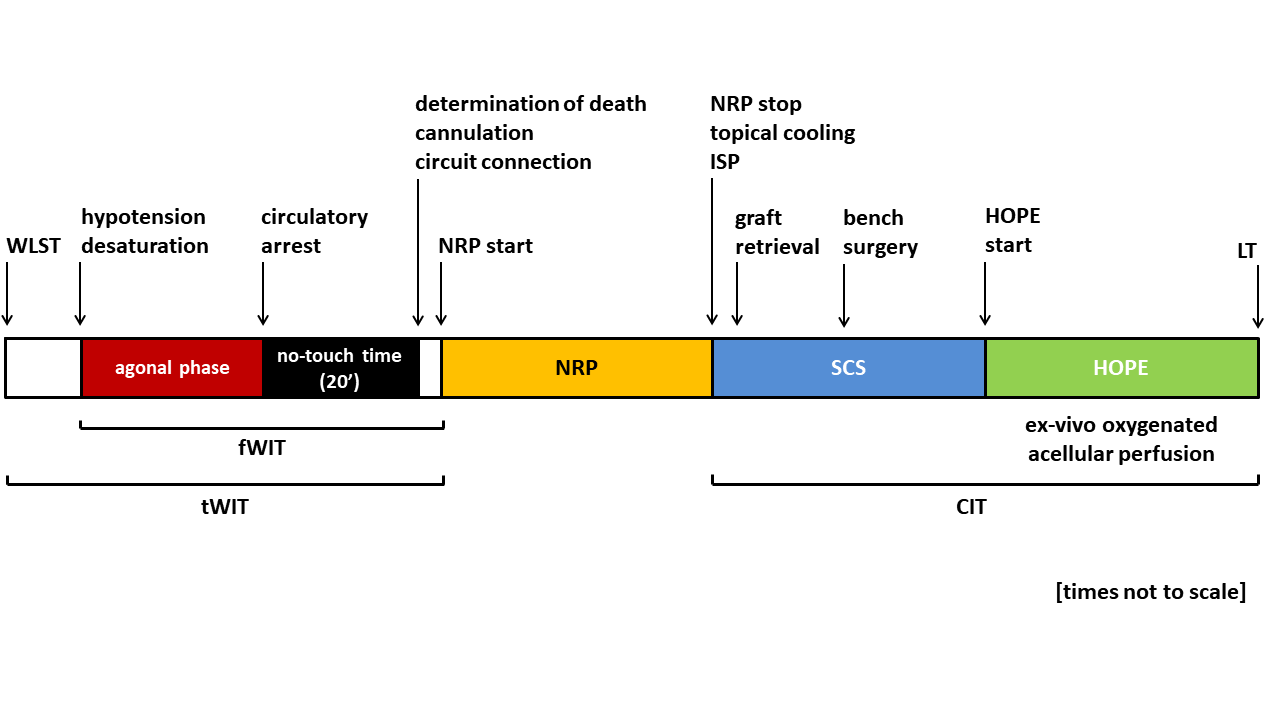

Supplement: Supplementary file 2 [file Image1.TIF]
